# Functional wiring of hypocretin and LC-NE neurons: implications for arousal

**DOI:** 10.3389/fnbeh.2013.00043

**Published:** 2013-05-20

**Authors:** Matthew E. Carter, Luis de Lecea, Antoine Adamantidis

**Affiliations:** ^1^Department of Biochemistry, University of WashingtonSeattle, WA, USA; ^2^Department of Psychiatry and Behavioral Sciences, Stanford UniversityStanford, CA, USA; ^3^Department of Psychiatry, Douglas Mental Health University Institute, McGill UniversityMontreal, QC, Canada

**Keywords:** hypocretin, orexin, hypothalamus, neural circuits, optogenetics, arousal system, sleep, norepinephrine

## Abstract

To survive in a rapidly changing environment, animals must sense their external world and internal physiological state and properly regulate levels of arousal. Levels of arousal that are abnormally high may result in inefficient use of internal energy stores and unfocused attention to salient environmental stimuli. Alternatively, levels of arousal that are abnormally low may result in the inability to properly seek food, water, sexual partners, and other factors necessary for life. In the brain, neurons that express hypocretin neuropeptides may be uniquely posed to sense the external and internal state of the animal and tune arousal state according to behavioral needs. In recent years, we have applied temporally precise optogenetic techniques to study the role of these neurons and their downstream connections in regulating arousal. In particular, we have found that noradrenergic neurons in the brainstem locus coeruleus (LC) are particularly important for mediating the effects of hypocretin neurons on arousal. Here, we discuss our recent results and consider the implications of the anatomical connectivity of these neurons in regulating the arousal state of an organism across various states of sleep and wakefulness.

Sleep and wakefulness are two mutually exclusive states that cycle with both ultradian and circadian periods throughout the animal kingdom. Wakefulness is a conscious state in which an animal can perceive and interact with its environment. After prolonged period of wakefulness, sleep pressure increases and leads to the onset of sleep that is characterized as a period of relative inactivity with stereotyped posture and higher sensory threshold.

In mammals, sleep is generally divided into slow-wave sleep (SWS, or NREM sleep in humans), and rapid eye movement (REM) sleep (also called “paradoxical sleep”). Wakefulness, SWS and REM sleep are distinct behavioral states that can be defined by precise electroencephalographic (EEG) and electromyographic (EMG) features. During wake, low-amplitude, mixed-frequency oscillations predominate. SWS is characterized by high-amplitude slow oscillations (0.5–4 Hz) whose predominance (as measured by the EEG power density) reflects the depth of sleep. REM sleep is a singular behavioral state, characterized by faster, mixed frequencies oscillations, among which theta (5–10 Hz) oscillations dominate in rodents, accompanied by muscle atonia, as well as fluctuation of the heart and breathing rates.

Although states of sleep and wakefulness are qualitatively and quantitatively easy to characterize, it is surprisingly difficult to define what is meant by “arousal.” The term arousal usually refers to the degree of vigilance and alertness during wakefulness, manifesting as increased motor activation, responsiveness to sensory inputs, emotional reactivity, and enhanced cognitive processing.

The brain mechanisms underlying the organization of the sleep-wake cycle and general level of arousal remain unclear and many classical studies have identified several populations of neurons whose activity correlates with distinct behavioral states. It was originally assumed that neurons that are active before behavioral transitions (i.e., neurons active preceding a sleep-to-wake transition) *promote* the coming state, while neurons that are active during a specific state (wakefulness or sleep) are important to *maintain* it. This view is made more complicated by the understanding that neurons in a network may show state-boundary-associated activity because of connectivity to other, more causal neurons without being directly responsible themselves for state transitions. Nevertheless, it has generally been inferred that there are neural populations that play a causal role in sleep and/or arousal states. Populations that are thought to promote arousal include: the hypocretin (hcrt—also called “orexins”)-expressing neurons in the lateral hypothalamus, the noradrenergic locus coeruleus (LC)-expressing neurons in the brainstem, the serotoninergic dorsal raphe nuclei (DRN) in the brainstem, the histaminergic tuberomammilary nucleus (TMN) in the posterior hypothalamus, the cholinergic pedunculopontine (PPT) and laterodorsal tegmental (LDT) nuclei in the midbrain, as well as cholinergic neurons in the basal forebrain (Jones, 2003). In contrast, inhibitory neurons from anterior hypothalamic structures are active during SWS, while Melanin-Concentrating Hormone (MCH) neurons from the lateral hypothalamus, as well as glutamatergic and GABAergic neurons from the brainstem are active during REM sleep (Fort et al., 2009).

In recent years, we and others have begun using optogenetic technology with various mouse models to address questions such as *How do arousal systems regulate wakefulness and arousal*? *How do they functionally interact to promote, maintain or broaden arousal in specific contexts*? In our recent studies, we have been particularly interested in neurons that express hcrt (de Lecea et al., 1998; Sakurai et al., 1998). The hcrt are two neuro-excitatory peptides (de Lecea et al., 1998; Sakurai et al., 1998) produced in ~3200 neurons in the mouse lateral hypothalamus (~6700 and 50,000–80,000 in the rat and human brain, respectively) (de Lecea and Sutcliffe, 2005; Modirrousta et al., 2005). These neurons receive functional inputs from multiple systems distributed in the cortex, limbic system, sub-cortical areas including the hypothalamus itself, thalamus, and ascending projections from the brainstem cholinergic nuclei, the reticular formation, the midbrain raphe nuclei, and the periaqueductal gray. In turn, these neurons project throughout the central nervous system, including to arousal and reward centers of the brain, to neurons expressing hcrt receptors (OX1R and OX2R). The afferent and efferent projections of hcrt neurons suggest that they play a role in multiple hypothalamic functions including regulating the sleep/wake cycle and goal-oriented behaviors. Interestingly, we have found that a specific efferent projection from hcrt neurons to noradrenergic LC neurons mediate sleep-to-wake transitions and possibly more general aspects of arousal.

Here, we summarize recent optogenetic experiments that test the hypothesis that hcrt and LC neurons cause arousal state transitions and maintenance (Adamantidis et al., 2007; Carter et al., 2009, 2010, 2012). First, we briefly highlight and summarize previous reports about these systems using traditional genetic and pharmacological techniques. Next, we integrate our own findings using optogenetic probes to selectively stimulate or inhibit these systems in freely-moving mice. Finally, we discuss unresolved questions and speculate on future anatomical and functional dissections of arousal circuits.

## Hypocretins, wakefulness, and narcolepsy

hcrt neurons are generally silent during quiet wakefulness, SWS, and REM sleep but show high discharge rates during active wake and REM sleep-to-wake transitions (Lee et al., 2005; Mileykovskiy et al., 2005; Takahashi et al., 2008; Hassani et al., 2009). In addition, they show high discharge rates during arousal elicited by environmental stimuli (e.g., an auditory stimulus) (Takahashi et al., 2008) and goal-oriented behavior (Mileykovskiy et al., 2005; Takahashi et al., 2008). These studies suggest that hcrt neurons participate to sleep-to-wake transitions, as well as in the increased alertness observed during various goal-oriented behaviors.

Blockade or suppression of hcrt signaling demonstrates the necessity of hcrt for the integrity of behavioral states in mice, rats, dogs, humans, and possibly zebrafishes (Sakurai, 2007; Yokogawa et al., 2007). Indeed, the most compelling loss-of-function evidence comes from the link between hcrt deficiency and the symptoms of narcolepsy (Peyron et al., 2000; Saper et al., 2010). Narcoleptic patients with cataplexy have a complete absence of *hcrt* gene transcripts in the hypothalamus as well as non- or barely-detectable levels of hcrt in the cerebrospinal fluid (Thannickal et al., 2000; Sakurai, 2007; Yokogawa et al., 2007). Doberman narcoleptic dogs bear a mutation in *OX2R*, and all genetically engineered rodents with either a deletion of *hcrt*, *OX2R*, or hcrt cells present behavioral arrests that resemble cataplexy, the hallmark of narcolepsy (Jones, 2003; Sakurai, 2007; Sehgal and Mignot, 2011). Importantly, genetic rescue of *hcrt* gene expression alleviated narcolepsy symptoms in mice (Liu et al., 2011; Blanco-Centurion et al., 2013).

Intracerebroventricular (i.c.v.) infusion of hcrt peptides or hcrt agonists causes an increase in the time spent awake and a decrease in SWS and REM sleep [review in Sakurai (2007)]. Stereotactic injection of the peptide in the LC, LDT, basal forebrain, or the lateral hypothalamus increased wakefulness and locomotor activity often associated with a marked reduction in SWS and REM sleep (Hagan et al., 1999). More recently, genetic dis-inhibition of hcrt neurons using a selective GABA-B receptor gene deletion only in hcrt neurons induced severe fragmentation of sleep/wake states during both the light and dark periods without showing an abnormality in total sleep/wake durations or signs of cataplexy (Matsuki et al., 2009). Collectively, these data suggest that the hcrt peptides are important to define boundaries between sleep and wake states, as shown by the fragmentation of sleep and wake state in animal models of narcolepsy.

Although it is widely documented that the biological function of hcrt peptides is necessary to maintain appropriate arousal and sleep, it remains unclear which of the two hcrt receptors, OX1R, or OX2R, is biologically responsible for hcrt's effects on arousal, as well as sleep stability and muscle tone control. *OX1R* mRNA is expressed in many brain areas, in particular the LC, raphe nuclei, LDT while *OX2R* mRNA shows a complementary pattern of expression in cerebral cortex, raphe nuclei, as well as dorsomedial and posterior (in the tuberomammillary nucleus) hypothalamus (Trivedi et al., 1998; Marcus et al., 2001; Mieda et al., 2011). Thus, it has been proposed that the control of wakefulness and NREM sleep-to-wake depends critically on OX2R (Mochizuki et al., 2011) while the dysregulation of REM sleep (unique to narcolepsy-cataplexy) results from loss of signaling through both OX1R and OX2R (Mieda et al., 2011). However, their implications in the regulation of narcolepsy, in particular cataplexy and sleep attack, remain unclear. Dogs with heritable narcolepsy bear a null mutation in the *OX2R* gene (Lin et al., 1999) and the corresponding mouse model, the *OX2R* KO mice, show less severe symptoms than the dogs (Willie et al., 2003). Although OX1R participates to the regulation of arousal (Mieda et al., 2011), its contribution to the symptoms of narcolepsy remains to be further characterized.

Importantly, activity in other arousal systems is strongly perturbed during cataplexy. LC neurons cease discharge (Gulyani et al., 1999) and serotoninergic neurons significantly decrease their activity (Wu, 2004), while cells located in the amygdala (Gulyani et al., 2002) and the TMN showed an increased level of firing (John et al., 2004). This association suggests that both OX1R (LC, raphe), and OX2R (TMN, raphe) are involved in the maintenance of appropriate muscle tone. Recent studies also highlighted the role of altered cholinergic systems in triggering cataplexy in narcoleptic mice (Kalogiannis et al., 2011, 2010). Therefore, an important, unresolved goal is to identify the functional wiring of hcrt neurons, as well as the dynamics of synaptic release from hcrt terminals to precisely delineate the downstream projections (de Lecea et al., 2012) that control arousal, sleep states, muscle tone, and goal-oriented behaviors.

## The locus coeruleus, norepinephrine, and arousal

The LC is adjacent to the 4^th^ ventricle in the brainstem and contains neurons that synthetize the monoamine norepinephrine (NE). Although four other cell populations also produce NE (the A1, A2, A5, and A7 cell groups), the LC produces ~50% of the brain's total NE and is the only source to the cortex. There are many functional NE receptors located throughout the brain, with α1 and β receptors usually causing excitatory postsynaptic potentials and α2 receptors usually causing inhibitory postsynaptic potentials. α2 receptors are densely found on LC neurons (Berridge and Waterhouse, 2003) themselves and serve as inhibitory autoreceptors to suppress intrinsic activity.

Recordings in awake behaving animals show that LC neurons fire tonically at 1–3 Hz during awake states, fire less during SWS sleep, and are virtually silent during REM sleep (Aston-Jones and Bloom, 1981; Jones, 2003; Saper et al., 2010). The LC also fires phasically in short bursts of 8–10 Hz during the presentation of salient stimuli that may increase wake duration. Like hcrt neurons, alterations in discharge rate precede changes in sleep-to-wake transitions (Aston-Jones and Bloom, 1981), suggesting that these cells are important for transitions to wakefulness or attention.

Interestingly, physical lesions of the LC do not elicit consistent changes in cortical EEG or behavioral indices of arousal (Lidbrink, 1974; Blanco-Centurion et al., 2007). Genetic ablation of dopamine beta-hydroxylase, an enzyme required for NE synthesis, also does not disrupt sleep-wake states (Hunsley et al., 2006). This suggests the presence of redundant neural circuitry, external to the LC structure, supporting cortical activity and compensatory developmental mechanisms, respectively. However, central injections of pharmacological antagonists of α1 and β noradrenergic receptors (Berridge and España, 2000) or agonists of inhibitory α2 autoreceptors (De Sarro et al., 1987) have substantial sedative effects. Central administration of NE directly into the ventricles or forebrain promotes wakefulness (Segal and Mandell, 1970; Flicker and Geyer, 1982). Stimulation of neurons in the LC using local microinjections of a cholinergic agonist (bethanechol) produces rapid activation of the forebrain EEG in halothane-anesthetized rats (Berridge and Foote, 1991). Recently, the LC-NE system was shown to be critical for maintaining the increased membrane potential of cortical neurons in awake compared to sleep states (Constantinople and Bruno, 2011). Taken together, these studies imply that the LC-NE system desynchronizes cortical activity and increases cortical membrane potential to increase arousal.

## Optogenetic dissection of hcrt and LC-NE control of arousal

The activity of hcrt and LC-NE neurons correlates with sleep-to-wake transitions, however, it has been difficult to selectively stimulate or inhibit specific hcrt and LC-NE populations with a temporal resolution relevant to sleep or wakefulness episodes, and to achieve spatial selectivity to probe those cells without affecting surrounding cells or fibers-of-passage. In an effort to better understand the temporal dynamics of neural circuits of wakefulness, we recently applied optogenetics to reversibly and selectively manipulate the activity of hcrt and LC neurons in freely-moving animals (Adamantidis et al., 2007; Carter et al., 2009, 2010, 2012). Optogenetics uses actuator opsin molecules (e.g., channelrhodopsin-2 (ChR2) or halorhodopsin—NpHR) to selectively activate or silence genetically-targeted cells, respectively, with flashes of light at specific wavelength (Boyden et al., 2005). Further information about optogenetic technology can be found in many other excellent reviews (Zhang et al., 2006; Miesenbock, 2009; Scanziani and Häusser, 2009; Yizhar et al., 2011; Deisseroth, 2012).

**Figure 1:**
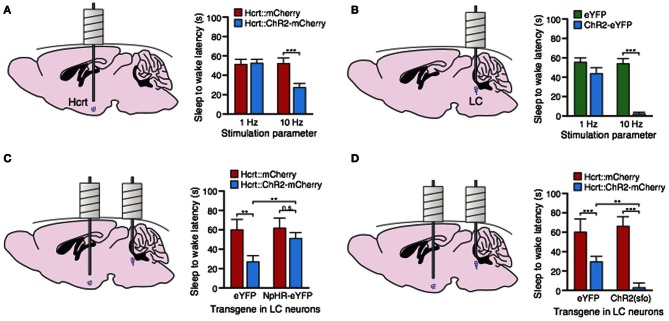
**Optogenetic dissection of arousal circuits of the brain. (A)** Stimulation of hcrt neurons with ChR2 causes a decrease in sleep-to-wake latency at 10 Hz but not 1 Hz (data from Adamantidis et al., 2007). **(B)** Stimulation of LC neurons with ChR2 causes immediate sleep-to-wake transitions at 10 Hz (data from Carter et al., 2010). **(C)** Stimulation of hcrt neurons at 10 Hz fails to decrease sleep-to-wake latencies when the LC is concomitantly inhibited with NpHR (data from Carter et al., 2012). **(D)** Stimulation of the LC with a mutated version of ChR2 called a step-function-opsin (sfo) that increases membrane excitability enhances hcrt-mediated sleep-to-wake transitions (data from Carter et al., 2012). ^**^P < 0.01; ^***^P < 0.0001.

To deliver these actuators to hcrt or LC neurons, we used lentiviral and cre-dependent adeno-associated viral (AAV) gene delivery tools, respectively, under the control of cell-type specific promoters (Adamantidis et al., 2007). To deliver light to the hcrt or LC field, we designed optical-neural interfaces in which optical fibers were chronically implanted on the mouse skull, as described elsewhere (Adamantidis et al., 2005, 2007; Aravanis et al., 2007; Zhang et al., 2010). Using this strategy, we were able to control hcrt neural activity both *in vitro* and *in vivo* with millisecond-precise optical stimulation (Adamantidis et al., 2007). The high temporal and spatial precision of stimulation allowed us to mimic the physiological range of hypocretin neuron discharge rates (1–30 Hz) (Hassani et al., 2009). Indeed, we used light pulse trains for our optogenetic stimulation that were based on parameters on the actual frequency analysis of hcrt neurons *in vivo* (this is also true for optogenetic control of LC-NE neurons described below). We found that direct unilateral optical stimulation of hcrt neurons increased the probability of transitions to wakefulness from either SWS or REM sleep (Figure 1A). Interestingly, high frequency optical stimulation (5–30 Hz light pulse trains) reduced the latency to wakefulness whereas 1 Hz trains did not, suggesting a frequency-dependent synaptic release of neurotransmitter (glutamate) and neuromodulators, including hcrt or dynorphin from the terminals. We further showed that the effects of stimulating hcrt neurons could be blocked by injection of a OX1R antagonist or by genetic deletion of the hcrt gene, suggesting that hcrt peptides mediate, at least in part, optogenetically-induced sleep-to-wake transitions. These results show that hcrt release from hcrt-expressing neurons is necessary for the wake-promoting properties of these neurons. Importantly, these results demonstrate a causal link between hcrt neuron activation and sleep-to-wake transitions, consistent with previous correlative studies. This was further supported by the fact that optical silencing of hcrt neurons promote SWS (Tsunematsu et al., 2011).

These results were recently confirmed by Sasaki and collaborators (Sasaki et al., 2011), who used a pharmacogenetic approach called Designer Receptors Exclusively Activated by Designer Drugs (DREADDs) to activate and suppress hcrt neural activity. DREADD technology allows bimodal modulation of neural activity with temporal resolution of several hours (Dong et al., 2010). They found that activation of hcrt neural activity increased wakefulness while suppression of hcrt activity promoted SWS.

In a second study (Carter et al., 2009), we demonstrated that hcrt control of sleep-wake transitions is under the dependence of sleep homeostasis processes since hcrt-mediated sleep-to-wake transitions are blocked by increased sleep pressure (caused by sleep deprivation). However, the effect of optogenetic stimulations of hcrt persisted in histamine decarboxylase knockout mice (mice that are unable to synthesize histamine) suggesting that another target that the histaminergic system is responsible for the effect of the hcrt. Finally, we showed that downstream arousal centers such as the LC neurons both increased their activity (as measured by c-Fos expression) in response to hcrt optogenetic stimulation. Because previous work showed an excitatory effect of hcrt on LC NE neurons (Bourgin et al., 2000), we investigated the hcrt-LC connection and focused our experimental investigations on the noradrenergic LC as a new target for optogenetic manipulation.

In a third study (Carter et al., 2010), we genetically targeted LC-NE neurons by stereotaxic injection of a Cre recombinase-dependent adeno-associated virus (rAAV) into knock-in mice selectively expressing Cre in tyrosine hydroxylase (TH) neurons (Atasoy et al., 2008; Tsai et al., 2009). We found that both NpHR and ChR2 were functional and could inhibit and activate, respectively, LC-NE neurons both *in vitro* and *in vivo* (Figure 1B). We found that optogenetic low frequency (1–10 Hz) stimulation of LC-NE neurons caused immediate (less than 5 s) sleep-to-wake transitions from both SWS and REM sleep. Stimulation of LC neurons during wakefulness increased locomotor activity and the total time spent awake, confirming the strong arousal effect. In contrast, NpHR-mediated silencing of LC-NE neurons decreased the duration of wake episodes but did not block sleep-to-wake transitions when animals were asleep. Taken together, this study showed that activation of LC-NE neurons is necessary for maintaining normal durations of wakefulness (NpHR experiment), and sufficient to induce immediate sleep-to-wake transitions, sustained wakefulness, and increased locomotor arousal. Thus, we proposed that the LC-NE neurons act as a fast tuning system to promote sleep-to-wake transitions and general arousal. Interestingly, we found that sustained optical activation of LC-NE neurons induces locomotor arrest (Carter et al., 2010). Such behavioral arrests share common symptoms with cataplexy, catatonia or behavioral freezing both in animal models and human patients (Scammell et al., 2009). Possible mechanisms may involve NE depletion from LC-NE synapse terminals or LC-NE overexcitation of brainstem motor nuclei that would lead to paralysis. Further study is required to unravel the underlying mechanisms.

In our most recent study (Carter et al., 2012), we tested the hypothesis that LC activity gates hcrt neuron's effects on sleep-to-wake transitions. Because hcrt and LC neural populations are located in distinct brain regions, it is physically possible to access both structures simultaneously in the same animal. We therefore took a dual optogenetic approach to stimulate hcrt neurons while concomitantly inhibiting or stimulating noradrenergic LC neurons during SWS sleep. We found that silencing LC neurons during hcrt stimulation blocked hcrt-mediated sleep-to-wake transitions (Figure 1C). In contrast, we found that increasing the excitability of LC neurons through step-function opsin (SFO) activation—which increase of target cells (Berndt et al., 2009)—during hcrt stimulation (using a LC stimulation protocol that by itself does not increase sleep-to-wake transitions) enhanced hcrt-mediated sleep-to-wake transitions (Figure 1D). Taken together, our results show that the LC serves as a necessary and sufficient downstream effector for hcrt-mediated SWS-to-wake transitions during the inactive period.

## hcrt and LC-NE system dynamics

Across our experimental studies, we observed that optogenetic manipulation of hcrt and LC-NE neurons affect sleep-to wake transitions with dramatically different temporal dynamics (Adamantidis et al., 2007; Carter et al., 2009, 2010, 2012). Acute optical activation of hcrt neurons causes sleep-to-wake transitions over a time period of 10–30 s, while stimulation of LC neurons causes sleep-to-wake transitions in less than 5 s. One explanation is that hcrt neurons may act as an upstream integrator of arousal during hypothalamic-related functions while the LC-NE system acts as a primary effector for arousal, stress, and attention. However, the neuronal effector systems are likely redundant and activated by distinct sets of inputs. Therefore, we cannot rule out that blocking other arousal systems, such as the central histaminergic and cholinergic systems, would also severely affect hcrt-induced behavioral state transitions in other experimental conditions.

Besides these short-term effects, it is also interesting that sustained (i.e., semi-chronic) photostimulation experiments of ~1–4 h of hcrt neurons increased sleep-to-wake transitions without changing the total duration of wakefulness, whereas long-term photostimulation of LC-NE neurons significantly increased wakefulness duration. These results suggest that the hcrt system may regulate sleep-wake boundaries while LC-NE neurons may rather control wake duration by increasing cortical membrane potential and desynchronizing the cortical EEG.

The hypothalamic localization of hcrt neurons implies that these cells have a prominent arousal role during homeostatic processes, including sexual behavior, food foraging, stress response, and motivation. Besides their control of wakefulness, arousal systems also participate in reward-seeking behavior, sexual activity, flight-or-fight responses, etc. This redundancy may have consolidated arousal function across evolution and diversified brain mechanisms supporting wakefulness and arousal-related behaviors necessary for survival. For example, activation of the LC-NE system increases arousal and cause anxiety-like behaviors (Itoi and Sugimoto, 2010). In contrast, the neuropeptide S (NPS) system, a peptide produced by a restricted neuronal population ventral to the LC, also increases arousal but *decreases* anxiety (Pape et al., 2010). Thus, to support such diverse behavioral functions, arousal circuits must have reached a high level of specification, possibly through a selective compartmentalization of their afferent and efferent connections, transmitters/modulators release capabilities and coherent activity with others arousal circuits.

## Perspectives

In the past five years, the combination of optogenetics, genetically-engineered mouse models, and EEG/EMG analysis of sleep has provided a unique and powerful set of tools to further understand the contributions of the hcrt and LC systems to arousal, as well as other populations of neurons that regulate degrees of sleep and wakefulness. Targeting optogenetic probes to other populations of neurons in the brain will determine their individual and combined roles in sleep/wake boundaries. Furthermore, these tools will allow us to determine the brain mechanism underlying wake states based on anatomical projections, synaptic neurotransmission, and dynamics of transmitter release. The ability to target and selectively manipulate these circuits with high temporal precision (<1 s) further allows the possibility to investigate their role in a wide spectrum of behaviors such as food intake, addiction, stress, attention, and sexual arousal. Ultimately, these studies may unravel the pathophysiological mechanisms of psychiatric disorders such as chronic anxiety, addiction, attention deficit, and depression.

### Conflict of interest statement

The authors declare that the research was conducted in the absence of any commercial or financial relationships that could be construed as a potential conflict of interest.
